# Evaluation of head posture in patients with temporomandibular joint disorders: a cross-sectional study

**DOI:** 10.1590/1806-9282.20241474

**Published:** 2025-05-02

**Authors:** Elif Esra Ozmen, Bayram Sonmez Unuvar

**Affiliations:** 1 Karamanoglu Mehmetbey University, Department of Oral and Maxillofacial Surgery – Karaman, Turkiye.; 2 Konya Ticaret Odası Karatay University, Faculty of Health Sciences, Department of Audiology – Konya, Turkiye.

**Keywords:** Jaw diseases, Pain, Posture, Temporomandibular joint disorders

## Abstract

**OBJECTIVE:**

This study evaluated head posture in patients with temporomandibular joint disorders and explored the effects of postural changes on clinical parameters.

**METHODS:**

In total, 81 individuals diagnosed with temporomandibular joint disorders participated in this cross-sectional prospective study. Demographics, pain status, head posture, and jaw movement data were collected. Head posture was assessed using the Posture Screen Mobile application.

**RESULTS:**

Results indicated moderate negative correlations between pain and mouth opening (rho=-0.437, p<0.001) and maximum mouth opening (rho=-0.427, p<0.001). Anterior translation showed weak positive correlations with mouth opening and maximum mouth opening, while right lateral translation exhibited a weak positive correlation with pain (rho=0.264, p=0.017). Posterior angulation showed weak significant correlations with pain, mouth opening, and maximum mouth opening.

**CONCLUSION:**

These findings suggest that head posture has a significant influence on temporomandibular joint disorder symptoms. Treatment strategies addressing postural abnormalities may help alleviate symptoms and enhance the quality of life in temporomandibular joint disorder patients.

## INTRODUCTION

The temporomandibular joint (TMJ) is a critical structure that enables essential functions such as chewing, speaking, and swallowing by coordinating complex jaw movements. TMJ disorders (TMD) are a group of functional impairments characterized by symptoms such as pain, restricted jaw movement, joint sounds, and muscle spasms. TMD significantly impacts patients’ quality of life, often requiring a multidisciplinary approach for effective management^1,2^.

Head posture plays a crucial role in maintaining musculoskeletal balance and proper body mechanics. Deviations in normal head posture, such as forward head posture or lateral translations, have been linked to various health issues, including neck pain, headaches, and even TMD^3,4^. Abnormal head posture is thought to exacerbate TMD symptoms by increasing strain on the TMJ and associated muscles^4,5^.

Previous studies have explored the relationship between TMD and head posture, suggesting that postural deviations may worsen TMD symptoms^6,7^. However, the existing literature presents inconclusive findings, necessitating further research to clarify the impact of head posture on TMD. Understanding this relationship is critical for developing comprehensive treatment strategies that not only target the TMJ but also address postural abnormalities.

This study aims to evaluate head posture in patients with TMD and investigate the effects of these postural deviations on clinical symptoms such as pain and jaw movement limitations. By understanding this relationship, clinicians can develop targeted interventions to alleviate symptoms by correcting postural imbalances. We hypothesize that there is a significant relationship between head posture deviations, levels of pain, and the range of jaw movements in patients with TMD. Specifically, we expect that abnormal head posture will correlate with increased pain levels and restricted jaw mobility. Therefore, addressing postural abnormalities may help alleviate TMD symptoms and improve overall patient outcomes.

## METHODS

### Study design

This research was conducted as a cross-sectional prospective study. Approval for the study was granted by the Karamanoglu Mehmetbey University Faculty of Medicine Clinical Research Ethics Committee (Decision No. 05-2024/20). The study took place between May and June 2024 at the faculty hospital, involving 81 volunteer participants diagnosed with TMD. All procedures were carried out following the principles of the Declaration of Helsinki. This study was reported in accordance with the STROBE statement.

### Participants

In total, 81 participants aged between 18 and 65 years, diagnosed with muscle-related TMD, were included in the study. Patients presenting with jaw pain, ear pain, or joint sounds were evaluated and diagnosed using the Research Diagnostic Criteria for Temporomandibular Disorders (RDC/TMD) developed by Dworkin and Leresche^8^. Exclusion criteria included participants with pre-existing skeletal disorders, intra-articular TMD, tumors, infections, psychiatric diagnoses, systemic diseases, bleeding disorders, fibromyalgia, and uncooperative behavior. All participants provided written informed consent before participation, and confidentiality was ensured by using unique identifiers for each participant.

**Figure 1:**
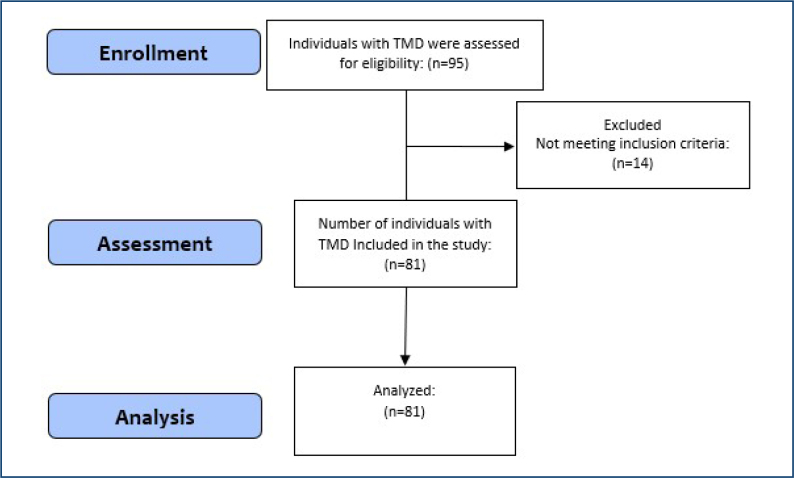
Flow diagram.

A pilot study was conducted with 10 participants to determine the necessary sample size. Using the G*Power software (version 3.0.10, Franz Faul, University of Kiel, Germany), the effect size was calculated as r=0.31, based on the pilot results. A minimum of 73 participants was needed to achieve 80% power at a 0.05 significance level (α) and a type II error rate (β) of 0.20. To account for potential data loss, an additional 10% of participants were included, resulting in a total sample size of 81 participants (Figure 1).

### Outcome measures

#### Sociodemographic information

Data on participants’ age, gender, education level, income level, marital status, joint sounds during mouth opening and closing, protrusion levels, tooth wear, night guard use, ear pain, jaw locking, headaches, and occlusion angle were collected.

#### Pain

The Visual Analog Scale (VAS) was used to measure participants’ pain levels. The VAS consists of a 10 cm line, with 0 representing "no pain" and 10 representing "the most severe pain imaginable." Participants marked their pain intensity on this line for both resting and functional states^9^.

#### Posture

Posture was evaluated using the Posture Screen Mobile (PSM) (PostureCo Inc., Trinity, FL, USA) application, which is a validated and reliable tool for postural analysis. Calibration of the PSM was performed before each session to ensure accuracy. Participants were photographed from the front, back, right, and left lateral views while standing in a standardized position. Postural deviations were calculated using the craniovertebral angle, determined by the angle between the horizontal axis and a line connecting the tragus of the ear to the C7 spinous process. Parameters such as anterior and posterior translations, lateral translations, and angulations were assessed^10,11^.

### Jaw motions

Jaw movement measurements included maximum mouth opening, mouth opening, and lateral movements. Maximum mouth opening was defined as the maximum distance between the upper and lower incisors in millimeters. Lateral movements were measured using a digital caliper, which recorded the distance the jaw could move to the right and left from the midline^12^.

### Statistics

Statistical analyses were performed using IBM SPSS version 27 (IBM Corp., Armonk, NY, USA). Data normality was assessed using the Kolmogorov-Smirnov test and histogram analysis. Due to the non-normal distribution of the data, Spearman correlation tests were used to evaluate the relationships between pain, maximum mouth opening, and various postural parameters. Significance was set at p<0.05. Spearman correlation coefficients (r) were interpreted as follows: 0–0.19 very weak, 0.2–0.39 weak, 0.40–0.59 moderate, 0.6–0.79 strong, and 0.8–1 very strong^13^.

## RESULTS

In total, 81 participants, ranging in age from 22 to 58 years, were included in the study. Sociodemographic information for the participants is presented in Table 1.

**Table 1:** Participants’ sociodemographic information and clinical parameters.

		n (%)	
Gender	Male	35 (43.2)	0.222
	Female	46 (56.8)	
Marital status	Married	36 (44.4)	0.317
	Single	45 (55.6)	
Education level	Primary school	2 (2.5)	<0.001*
	Middle school	15 (18.5)	
	High school	28 (34.6)	
	University	25 (30.9)	
	Graduate	11 (13.6)	
Income level	Low	33 (40.7)	<0.001*
	Medium	40 (49.4)	
	High	8 (9.9)	
Sound during mouth opening	Yes	72 (88.9)	<0.001*
	No	9 (11.1)	
Protrusion	1	3 (3.7)	<0.001*
	2	47 (58.0)	
	3	31 (38.3)	
Tooth wear	No wear	24 (29.6)	<0.001*
	Enamel	50 (61.7)	
	Dentin	7 (8.6)	
Night guard	Yes	19 (23.5)	<0.001*
	No	62 (76.5)	
Ear pain	Yes	50 (61.7)	0.035*
	No	31 (38.3)	
Headache	Yes	50 (61.7)	0.035*
	No	31 (38.3)	
Jaw locking	Yes	31 (38.3)	0.035*
	No	50 (61.7)	
Occlusion angle	1	29 (35.8)	0.010*
	2	37 (45.7)	
	3	15 (18.5)	

Chi-square test,

* p<0.05.

n: number of participants; %: percentage.

In this study, the relationships between various postural parameters and pain, mouth opening, and maximum mouth opening in patients with TMD were analyzed using Spearman correlation tests. According to the analysis results, a moderate negative and significant correlation was found between pain and mouth opening (rho=-0.437, p<0.001). Similarly, a moderate negative and significant relationship was identified between pain and maximum mouth opening (rho=-0.427, p<0.001).

A weak negative and significant relationship was found between anterior translation and pain (rho=-0.239, p=0.032). Additionally, weak positive and significant relationships were found between anterior translation and mouth opening (rho=0.224, p=0.044) and maximum mouth opening (rho=0.335, p=0.002).

No significant correlation was found between anterior angulation and the examined parameters. Right lateral translation showed a weak positive and significant correlation with pain (rho=0.264, p=0.017). Furthermore, weak negative significant relationships were found between right lateral translation and maximum mouth opening (rho=-0.233, p=0.036) and anterior translation (rho=-0.396, p<0.001). Right lateral angulation showed a weak positive relationship only with right lateral translation (rho=0.232, p=0.037).

No significant correlation was found between posterior translation and the other examined parameters. Posterior angulation showed weak significant relationships with pain (rho=-0.239, p=0.032), mouth opening (rho=0.223, p=0.045), and maximum mouth opening (rho=0.259, p=0.020). Additionally, strong positive relationships were found with anterior translation (rho=0.524, p<0.001) and anterior angulation (rho=0.577, p<0.001). A weak negative correlation was observed with right lateral translation (rho=-0.416, p<0.001).

Left lateral translation showed weak positive relationships with anterior translation (rho=0.316, p=0.004), anterior angulation (rho=0.372, p<0.001), and posterior angulation (rho=0.343, p=0.002). Left lateral angulation exhibited weak positive relationships with left lateral translation (rho=0.298, p=0.007) and posterior angulation (rho=0.433, p<0.001) (Table 2).

**Table 2:** The relationships between various postural parameters and pain, mouth opening, and maximum mouth opening.

Variables	Pain	Mouth opening	Maximum mouth opening
Pain	–	0.437***	-0.427***
Mouth opening	-0.437***	–	0.948***
Maximum mouth opening	-0.427***	0.948***	–
Anterior translation	-0.239*	0.224*	0.335**
Anterior angulation	-0.115	0.084	0.128
Posterior translation	0.004	0.035	0.041
Posterior angulation	-0.239*	0.223*	0.259*
Right lateral translation	0.264*	-0.209	-0.233*
Right lateral angulation	0.004	-0.052	0.009
Left lateral translation	-0.164	0.155	0.172
Left lateral angulation	-0.049	0.061	0.075

Spearman correlation;

* p<0.05;

** p<0.01;

*** p<0.001.

## DISCUSSION

The findings of this study indicate that the majority of participants were women, which may suggest a higher prevalence of TMD among females. Previous research has explored the relationship between sex steroid receptors in masticatory muscles, considering factors such as age and gender, and emphasized the role of hormones in TMD^14^. Hormonal fluctuations in women can increase muscle tension and pain sensitivity, potentially exacerbating TMD symptoms. Thus, gender differences should be taken into account in the assessment and management of TMD.

A moderate negative and significant correlation was found between pain and mouth opening, as well as maximum mouth opening. This finding indicates that an increase in pain may lead to a decrease in mouth opening. The findings of this study are similar to the literature. The TMJ is associated with muscles and joint structures that control jaw movements, and dysfunction in these structures can lead to pain and restricted movement^15–17^. This aligns with previous research, such as the work by Minervini et al.^3^, which demonstrated that increased TMD-related pain significantly restricts mandibular mobility. Furthermore, pain-induced muscle guarding and spasms are well-documented phenomena that exacerbate joint stiffness and limit functional capacity^18^.

A weak negative relationship was found between anterior translation and pain. Additionally, weak positive relationships were found between anterior translation and mouth opening, as well as maximum mouth opening. The anterior translation is characterized by the forward displacement of the head, which can cause tension in the neck muscles, particularly the sternocleidomastoid and trapezius muscles. This tension can increase pressure on the TMJ, leading to pain and restricted movement. These findings are supported by studies in the literature^3,19^. Furthermore, forward head posture has been linked to altered biomechanics and increased strain on the cervical spine, which may further exacerbate TMD^6^. Corrective exercises aimed at improving head posture have shown promise in reducing TMD symptoms, highlighting the importance of addressing postural issues in clinical practice^20^.

The weak positive correlation between right lateral translation and pain suggests that lateral shifts in head posture may exacerbate pain symptoms. The lateral translation is characterized by the sideways displacement of the head, creating asymmetric loading on the neck muscles and spinal alignment. Lateral postural changes can cause asymmetric tension in the TMJ and associated muscle groups, leading to pain and dysfunction. This asymmetry can hinder the proper alignment of the TMJ, causing pain and restrictions in jaw movements^21^. Additionally, this misalignment can contribute to compensatory mechanisms in the cervical spine and shoulder girdle, further complicating the clinical picture^22^. Clinicians should consider incorporating postural assessment and correction into the treatment protocols for TMD patients, as addressing these imbalances can potentially alleviate pain and improve overall functional outcomes^3^.

The weak significant relationships between posterior angulation and pain, mouth opening, and maximum mouth opening indicate that posterior postural changes may also affect TMD symptoms. Posterior angulation is characterized by the backward tilt of the head, which can increase mechanical stress on the lower jaw and TMJ. This stress can lead to joint dysfunction and pain^23^. Such findings align with previous research indicating that abnormal head postures can contribute to altered mandibular dynamics and increased strain on the TMJ, exacerbating symptoms in participants with TMD^24^. Moreover, interventions focusing on correcting head posture have shown promising results in reducing TMJ pain and improving functional outcomes^3^. In addition, psychological factors like anxiety and stress may worsen TMD symptoms, as emotional stress can increase muscle tension and pain. Recent studies have shown that the COVID-19 pandemic, with its extended periods of social isolation, significantly affected individuals’ emotional well-being, leading to increased levels of anxiety, depression, and stress. Research indicates that these psychological factors were linked to greater TMD-related pain, particularly among vulnerable groups such as students^25^. Therefore, it is plausible that emotional stress could intensify the impact of postural deviations on TMD symptoms.

The findings of this study emphasize the importance of evaluating head posture in patients with TMD. It should be considered that postural deviations can exacerbate TMD symptoms and restrict jaw functions. Therefore, strategies to correct head posture should be developed in the treatment of TMD.

Although this study is a significant step in understanding the relationship between TMD and head posture, larger sample sizes and long-term studies are necessary. Future research should compare the effects of different treatment methods on head posture and TMD symptoms. Furthermore, the relationship between head posture and other musculoskeletal disorders should be explored. Specifically, the role of the neck and shoulder muscles and their impact on TMD should be investigated in detail.

This study has several limitations. The cross-sectional design limits the ability to establish causality, indicating the need for longitudinal studies to explore these relationships further. Additionally, while the study assessed the link between head posture, pain, and jaw movements, the long-term effects remain uncertain. Including a control group of healthy participants would have enhanced comparative analysis. The use of subjective measures, such as the VAS for pain, may introduce potential bias. Although clinical tools like RDC/TMD are commonly used, their diagnostic accuracy can sometimes fall short, whereas advanced imaging techniques, such as magnetic resonance imaging, may provide more precise diagnostics in complex TMD cases^26^.

## CONCLUSION AND RECOMMENDATIONS

This study is among the first to evaluate head posture in TMD patients using a mobile posture analysis application. The findings indicate that head posture is a significant factor influencing TMD symptoms, with deviations exacerbating pain and limiting jaw function. Integrating postural assessments into clinical practice can help identify high-risk patients and guide targeted treatment approaches. Correcting head posture through targeted treatment approaches can help reduce patients’ symptoms and improve their quality of life.
